# Comparison of Step Count Assessed Using Wrist- and Hip-Worn Actigraph GT3X in Free-Living Conditions in Young and Older Adults

**DOI:** 10.3389/fmed.2019.00252

**Published:** 2019-11-05

**Authors:** Stephane Mandigout, Justine Lacroix, Anaick Perrochon, Zdenek Svoboda, Timothee Aubourg, Nicolas Vuillerme

**Affiliations:** ^1^Université de Limoges, HAVAE, EA 6310, Limoges, France; ^2^Faculty of Physical Culture, Palacky University Olomouc, Olomouc, Czechia; ^3^Univ. Grenoble Alpes, AGEIS, Grenoble, France; ^4^Orange Labs, Meylan, France; ^5^Institut Universitaire de France, Paris, France

**Keywords:** activity tracker, step count, physical activity, older, free-living

## Abstract

**Background:** Walking represents a major component of physical activity (PA), and its restriction could degrade autonomy and quality of life. An important objective for preventive and/or rehabilitative strategies to improve balance and gait in normal and pathological aging conditions is to focus on physical activity. Activity monitors have recently been getting increasingly popular and represent a modern solution to measure—and communicate—PA notably in terms of steps/day. These activity monitors are well-suited for various populations as they can be worn on a variety of locations on the body, including the wrist and the hip (i.e., the two most common locations), in an undifferentiated way according to the manufacturer's instruction. The aim of this study was hence to verify potential differences in step count (SC) by comparing this parameter assessed using wrist- and hip-worn activity trackers over a 24-h period in free-living conditions in young and older adults.

**Methods:** Young adults (*n* = 22) and older adults (*n* = 22) voluntarily participated in this study. They were required to wear two commercially-available Actigraph GT3X+ activity monitors simultaneously at two locations recommended by the manufacturer, i.e., one positioned around the wrist and one above the hip, over a 24-h period in free-living conditions. The manufacturer's software was used to obtain estimates of the SC.

**Results:** For both groups, the wrist-worn activity tracker provided significantly higher SC than the hip-worn activity tracker did. For both placements on the body, older adults exhibited significantly lower SC than young adults. Interestingly, for both young and older participants, the difference between both measurements tended to decrease for longer distances.

**Conclusion:** The different estimations of the step count provided by the comparison between two identical Actigraph GT3x on the wrist or the hip during the 24-h observation period in free-living conditions in young and older adults strongly suggests that caution is needed when using total step per day values as an outcome to quantify walking behavior. Probably we can suggest the same caution across implementation of different activity Tracker.

## Introduction

Walking represents a major component of physical activity (PA), and its restriction could degrade autonomy and quality of life (1). An important objective for preventive and/or rehabilitative strategies to improve balance and gait in normal and pathological aging conditions is to focus on PA. Activity monitors have recently been getting increasingly popular and represent a modern solution to measure—and communicate—the amount of PA performed by its user (2).

A patient's level of PA can be estimated using either the daily energy expenditure or the step count (SC). PA recommendations are generally defined on the basis of energy expenditure (Kcal, MET.min^−1^, MET.h^−1^) (3). Unfortunately, the accuracy of the estimation of energy expenditure by activity trackers (AT) appears questionable regardless of the population (4). On the other hand, the SC is presented as a relatively stable and reliable indicator (2). Currently, no recommendation have been published regarding this indicator (2). Despite this, the SC may be used as a relevant indicator of a person's amount of PA, lifestyle (active vs. sedentary) and physical inactivity (2).

AT may be worn on a variety of locations on the body, including the ankle, the wrist, the hip, and around the neck. Depending on the model and manufacturer, some AT may be placed indiscriminately at different locations on the body (Actigraph GT3X for instance). The location of the AT must then be registered in the software for the device to correctly define the algorithm used to estimate the SC. In normal use, these algorithms are not publicly available because they are the property of the manufacturer. Moreover, fitting the device with a specific algorithm for its location implies that the same SC should be found regardless of the activity performed by the person.

The literature shows that the most accurate way to evaluate the SC under free-living conditions is to place the AT at the ankle (5, 6). However, for practical and aesthetic reasons, ATs are regularly placed on the hip or the wrist.

Incorrectly positioning the AT may alter its results due to the technology it relies on. Indeed, two main technologies are used based on the internal mechanism used to record steps, i.e., spring-suspended lever arm or accelerometer, among which accelerometry is increasingly used. Depending on the technology used and the position of the AT on the body, the resulting SC may be significantly different (2). Additionally, significant differences tend to appear depending on the type of activity, laboratory conditions (7, 8) or standardized activities (walking, running) (9–12), or free-living condition (5, 13), between the possible AT positions.

The Actigraph GT3X (Actigraph LLC, Penascola, FL, USA) represents the epitome of scientific accelerometers as it is unobtrusive, low-cost, and its sensitive triaxial accelerometers are capable of storing high-resolution, raw, unfiltered acceleration signals over long durations. The Actigraph monitor has been extensively studied in many situations: the validity for the evaluation of PA in healthy or pathological populations and the comparison with other AT (13–17); used as a gold standard in some studies (18, 19). Despite all these studies, the recent systematic review by Migueles et al. (20) conclude that it is necessary to take a cautious approach regarding the accuracy/reliability of Actigraph in estimating the SC in real-life situations as a function of 1-the mainly used positions (wrist and hip) and 2- the age of the subjects.

In order to better assess the accuracy according to the position or the type of AT, some studies have focused on the calculation of the absolute error rate (AER) (21). This calculation allows to determine whether wrist-hip differences could be attributed to a factor inherent to the AT or inherent to the subjects. Some studies have used this parameter in their experimental design (9, 19, 22). To the best of our knowledge, no study has addressed the relationship between the percentage of absolute error between the SC of the two prominent AT positions (hip and wrist) and the age of the wearer with the Actigraph GT3X. Within this context, the aim of this study was to compare the AER of SC assessed using wrist- and hip-worn Actigraph GT3X over a 24-h period in free-living conditions in young and older adults.

## Methods

### Study Population

Our study population was aged between 18 and 85 years, without medical contraindication, and volunteered to participate after signing a consent form. The exclusion criteria were: any cardiovascular pathologies or mobility issues. The sample was divided into two groups: a group of subjects aged 18–45 years and a group of subjects aged 70–85 years. The protocol was approved by the Comité d'Ethique pour les Recherches Non-Interventionnelles (CERNI) of the Grenoble-Alpes University, France. All subjects gave written informed consent in accordance with the Declaration of Helsinki.

### Materials

The material requirements for the study were two Actigraph GT3X accelerometers (ActiGraph Pensacola, FL, USA, www.actigraphcorp.com). These tri-axial accelerometers are used to record the SC along with various PA data. Accelerometer data were collected at a frequency of 80 Hz and aggregated to 60-s epochs for analyses. Following the manufacturer's guidelines (23), a Low Frequency Extension (LFE) filter was used to increases the device's sensitivity and detect low-frequency accelerations (i.e., slow walking).

### Experimental Design

This study was designed to record the PA of a sample in a free-living situation for 24 h. The AT were positioned as follows: one Actigraph GT3X at the hip (in the center of the pelvis) and a second one at the non-dominant wrist. Subjects were asked to remove the device before showers and for aquatic activities. To compare accelerometer data according to each location, we limited the data from all devices to the actual wearing time when both devices were worn. The Actigraph was placed in the morning (between 8 and 11 a.m.) and was picked up the next day at the same time. The registration period was programmed using the manufacturer's Actilife software v 6.13.3 (www.actigraphcorp.com/actilife/). A 24-h period of recording allowed us to avoid the risk of human failure (weariness, forgetfulness…). After verification by an investigator, the records appeared to be correct and usable.

The parameter used in this study was the SC. All equipment was activated before placing it on the previously described locations. The minimum required recorded duration of accelerometer data to be included in the analysis was 24 h for both AT. After the 24 h of recording, the subject was requested to return the equipment in order for the practitioner to transfer it using ActiLife and reset the devices for new use.

### Statistical Analysis

**Figure 1:**
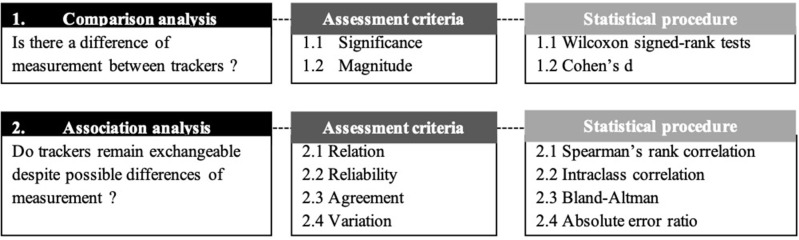
Synthesis diagram of the statistical treatment.

The step count data were presented in the form of mean and standard deviation. Firstly, to compare these data in free-living conditions in young and older adults according to trackers location, statistical tests of comparison were selected by testing step count data for normal distribution using the Shapiro-Wilk test. As the dependent variables did not conform to a Gaussian distribution, non-parametric comparative tests were chosen for the statistical analysis process. These tests were performed into two successive steps as follows (Figure 1):

(1) Comparison analysis. We compared the differences between the step counts provided by the wrist-worn and hip-worn trackers using two assessment criteria, namely the significance and the effect size of these differences. Significant differences were assessed by means of non-parametric Wilcoxon signed-rank tests. Effect sizes, also known as magnitude, were obtained using Cohen's d. This coefficient was calculated as a ratio of mean difference divided by mean standard deviation in both conditions. Effect sizes were considered small if d < 0.5, medium if 0.5 ≤ *d* < 0.8 and large with *d* ≥ 0.8 (24). We completed this statistical procedure by comparing the difference of measurement between hip-worn and wrist-worn AT according to the age category of the participants using Wilcoxon signed-rank tests.

(2) Association analysis. The results from step (1) were then complemented with an additional analysis to conclude whether trackers could remain exchangeable despite potential differences of measurement, and if so, to what extent. For this purpose, four assessment criteria were used, namely relation, reliability, agreement and variation. Relation between the step counts provided by the wrist-worn and hip-worn AT was calculated by means of Spearman's rank correlation coefficient rho. Reliability was measured by means of intraclass correlation coefficient (ICC). An ICC value between 0.00 and 0.40 was considered poor, 0.40 and 0.59 was fair, 0.60 and 0.74 was good, and 0.75 and 1.00 was excellent (25). The obtained scores were reported in Bland-Altman plots to visualize the agreement between the wrist-worn and hip-worn AT. Finally, for a comparison purpose, we assumed the hip location could be used as reference to study errors of measurements. To this end, we assessed the variation of the error measurement generated by the hip-worn AT according to the step count measured by the wrist-worn AT by calculating the absolute error ratio (AER). For each method, the absolute error for each estimated parameter [IC, FC, stride time (mean and CV), step time (mean and CV), and swing time (mean and CV)] was hence determined relatively to hip-worn trackers as follows:

(1) $$\begin{array}{r} AER=P-Pr \end{array}$$

Where pr is the reference value of the parameter p.

The level of significance was set as *p* < 0.05 in all statistical tests. All statistical calculations were completed using the R software environment (version 3.1.0; R Foundation for Statistical Computing, Vienna, Austria).

## Results

A total of 44 volunteers participated in this study. Participant characteristics are summarized in Table 1.

**Table 1:** Participant characteristics.

**(*n* = 44)**	**Young group (*n* = 22)**	**Older group (*n* = 22)**
Gender (male/female)	17/8	9/16
Hand dominance (right/left)	18/4	19/3
Age (years) : mean (*SD*)	27.2 (6.0)	76.6 (4.7)
Weight (kg) : mean (*SD*)	72.4 (13.0)	65.3 (10.1)
Height (cm) : mean (*SD*)	173.8 (7.9)	162.8 (7.5)

### Comparison Between the SC Provided by the Hip-Worn and by the Wrist-Worn AT

The SC for both AT are summarized in Table 2.

**Table 2:** Step count analysis.

	**All participants**	**Young participants**	**Old participants**
**Step count variable**			
Number of participants	44	22	22
Wrist-worn: mean (SD)	11,203 (4,543)	11,347 (3,258)	11,060 (5,787)
Hip-worn: mean (*SD*)	6,866 (4,655)	7,810 (3,969)	5,922 (5,172)
Wilcoxon test: *Z*-value; *p*-value	5.61; 1.43e-10	4.09; 9.53e-07	3.96; 1.57e-05
Regression coefficient^*^: α; β	5,671; 0.81	5,740; 0.72	5,711; 0.90
ICC (95% CI)	0.49 (0.23–0.68)	0.51 (0.12–0.76)	0.48 (0.09–0.87)
Mean of differences (95% limits of agreement)	4,337 (−1,314 to 9,987)	3,536 (−257 to 7,330)	5,137 (−1,632 to 11,906)
Wilcoxon test for the difference between young and older participants: *Z*-value; *p*-value		2.48; 0.0065	

*Descriptive statistics, comparison, correlation, agreement, and Bland-Altman parameters for the numbers of steps provided by the wrist-worn and hip-worn AT for all participants and by age group. SD, standard deviation; ICC, Intraclass coefficient correlation; CI, confidence interval*.

* *From the regression equation: M^wrist^ = α + β.M^hip^, where M^wrist^ is the wrist-worn measurements variable and M^hip^ is the hip-worn measurements variable*.

Regarding the overall population, measurements at the wrist were significantly higher than measurements at the hip with, respectively, 11,203 (*SD* = 4,543) vs. 6,866 (*SD* = 4,655) counted steps in average. A significant difference was also found in the young participants group, with, respectively, 11,347 (*SD* = 3,258) counted steps for wrist-worn AT vs. 7,810 (*SD* = 3,969) counted steps for hip-worn AT. Interestingly, this contrast increased for older participants: the mean SC provided by the wrist-worn AT was almost twice the value provided by the hip-worn devices, with, respectively, 11,060 (*SD* = 5,787) vs. 5,922 (*SD* = 5,172) counted steps in average. We then validated the significance of these measurement differences in the overall population, but also in young participants and older adults separately (*p*-values < 0.00001 for all three cases using Wilcoxon comparison tests, where the null hypothesis was a similarity between hip-worn and wrist-worn AT measurements). In addition, Cohen's d points out the strong effect size of this phenomenon for the overall population (*d* = 0.93), but also for both young (*d* > 1) and older participants (*d* > 0.8). We may however note that the error measurement between hip-worn and wrist-worn AT is significantly lower in young than older participants (*p*-value = 0.0065 and absolute *Z*-score = 2.48).

### Association Between SC Provided by Hip-Worn and Wrist-Worn AT

**Figure 2:**
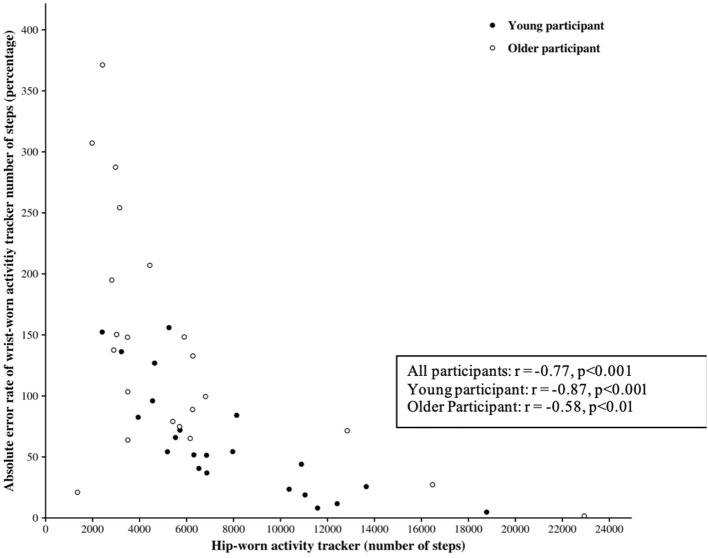
Wrist-worn and hip-worn AT step counts. Plot of wrist-worn against hip-worn AT measurements: all participants and by age group.

Correlation analyses showed significant positive relationships between the SC for wrist-worn and hip-worn AT in the overall population (Spearman's rho = 0.76, *p* < 0.001), for the young participants (Spearman's rho = 0.85, *p* < 0.001), and for the older participants (Spearman's rho = 0.70, *p* < 0.001) (Figure 2).

**Figure 3:**
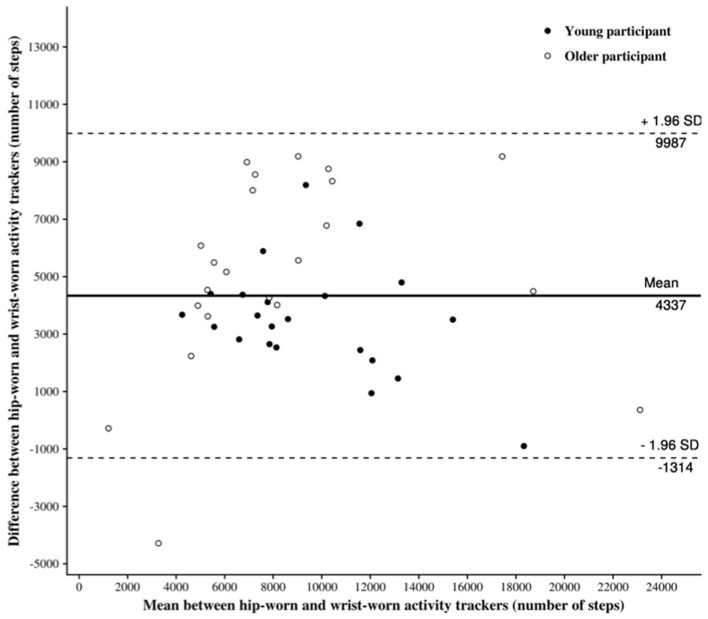
Bland-Altman plot of step count. Bland-Altman plot of differences between wrist-worn AT and hip-worn AT against the mean: all participants and by age group. The solid line represents the mean differences between the two measures obtained from the wrist-worn and the hip-worn AT; the dotted lines represent the limits of agreement (1.96 SDs).

The Bland-Altman plot for the SC measured for both AT positions is provided in Figure 3.

As indicated in Table 2, the estimated bias, i.e., the mean of the differences between the measurements of the wrist-worn and hip-worn AT, is 4,337 counted steps. This result implies that the wrist-worn AT tends to overestimate the SC in comparison to hip-worn AT.

### Variation of the Error Measurement

Finally, we assessed the error rate generated by the location of the AT in order to account for the possibility for this error to decrease at a certain threshold.

**Figure 4:**
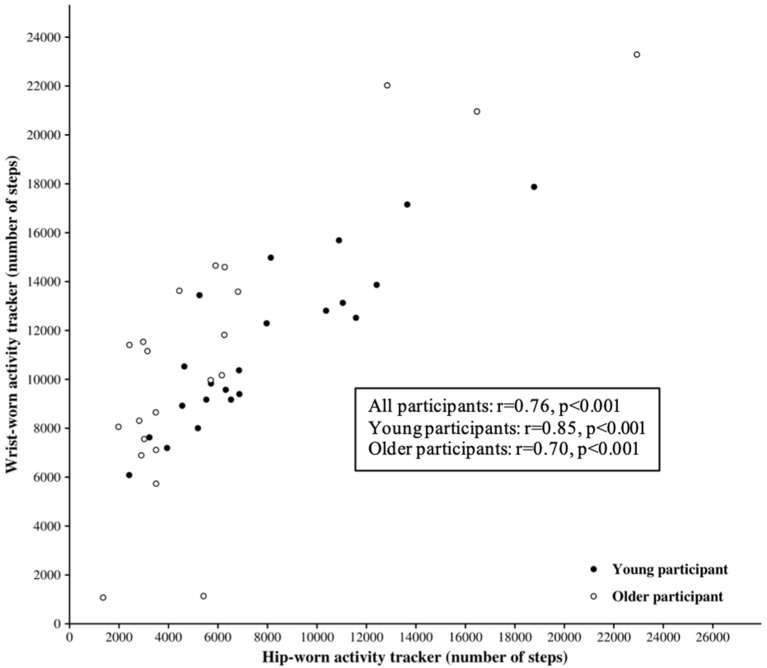
Absolute error rate (AER) of step count. AER between the step count provided by the wrist-worn activity tracker and by the hip-worn activity tracker. Here, $AER\left(M^{wrist},M^{Hip}\right)=\frac{\left|M^{wrist}-M^{Hip}\right|}{M^{Hip}}.100$, where is the wrist-worn measurements variable and *M*^*Hip*^ is the hip-worn measurements variable.

The absolute error rate (AER) between the SC provided by the wrist-worn AT and by the hip-worn AT is shown in Figure 4.

These results highlight a significant negative correlation between the AER and hip-worn SC in the overall population (Spearman's rho = −0.77, *p* < 0.001), in young participants (Spearman's rho = −0.87, *p* < 0.001) and in older participants (Spearman's rho = −0.58, *p* < 0.001). In other words, the error in the SC provided by the hip-worn AT tends to decrease according to the distance walked.

## Discussion

The objective of our study was to compare the absolute error rate (AER) and the SC assessed using wrist- and hip-worn Actigraph GT3X over a 24-h period in free-living conditions in young and older adults. Our results show that the more the individual walks during the day, the more the error in the SC provided by the hip-worn Actigraph GT3X tends to decrease. Our results further demonstrate an overestimation of the SC, as the SC measured by the Actigraph GT3X at the wrist is 39% higher than the SC measured at the hip (*p* < 0.05). Moreover, the hip-wrist difference is even more significant in older adults (*p* = 0.0065). Age could therefore be a factor influencing the measurement difference of the SC recorded by two Actigraph GT3X placed at different locations. To the best of our knowledge, no study has compared the SC difference given by identical Actigraph GT3X positioned at the hip and wrist during a 24 h recording in free-living conditions in young and old adults. Two recent studies conducted in young adults (13) and in older women (15) reported that the SC recorded by the Actigraph GT3X at the wrist was significantly higher than the number recorded at the hip when recording an activity in a real life situation. These results suggest a difference in the SC between the two positions, without any real indicators of the precision of one in relation to the other. The effect of age was however not assessed.

### Actigraph GT3X Accuracy Between Hip and Wrist According to Age

One of the parameters which could explain our results is the decrease of accuracy of AT when recording slow activities (11). Indeed, several studies demonstrate that few AT are capable of recording motion slower than 1 m.s^−1^ (26).

Older adults (60+) have been shown to self-select a walking speed of 1.18 m.s^−1^ (±0.17 m.s^−1^) (27) which can extend to 1.34 m.s^−1^ (±0.21 m.s^−1^) in healthy older adults (28). Webber et al. (17) carried out a comparative study (ActiGraph vs. Stepwatch, Hip vs. Ankle) during a hallway walk in 38 geriatric rehabilitation patients (83.2 ± 7.1 years of age), walking at a comfortable pace (0.4 m.s^−1^). Speeds under 1 m.s^−1^ are indeed commonly experienced in elderly populations and people with motor disabilities. In the study by Webber et al., the AER was low for slow walking speeds (<3%) and did not significantly differ between the StepWatch and the Actigraph GT3X+ (placed at the ankle); however, error values were higher (19–97%) when the Actigraph GT3X+ was worn at the hip during a hallway walk. In this study, the comparison involved two AT models, among which the Stepwatch was considered as the reference. In our study, the AER values were estimated at 39% between two Actigraph GT3X, positioned at the wrist and hip. Our results suggest that an activity monitor placed at the wrist can potentially overestimate low-speed activities and underestimate high-speed activities. According to Aziz et al. (29), wrist kinematics may represent a relatively small part of total body movements during walking (especially when walking with limited arm swing), and a relatively larger one during some sedentary activities such as simply moving hands while sitting.

Furthermore, Feng et al. (10) compared the accuracy of three commercially available accelerometers (Axivity AX3, Actigraph GT3X, and APDM-Opal) and pointed out the importance of customizing the AT placement and algorithms to maximize the measurement accuracy when selecting accelerometers specifically designed to measure the SC for slower walking speeds. Unfortunately, these algorithms are proprietary information and cannot be accessed by the standard user. Despite the accessibility of information, the Actigraph implements a Low Frequency filter (LFE) in the Actilife software. A normal filter can detect accelerations within a frequency range of 0.25–2.5 Hz, while the LFE filter establishes a lower threshold to capture slower movements. Despite this filter, the results vary drastically depending on the body positions (20).

### SC as a Function of at Locations on the Body

The lower SC provided by the Actigraph GT3X placed at the hip tended to generate greater absolute error between both positions (hip vs. wrist). The traditional AT was designed to be worn at the hip, attached to a belt or waistband (2). Many studies demonstrate that the accuracy of the measurement of the SC would increase when the AT is worn on the hip compared to the wrist during standardized activities such as walking and running (9, 13, 30). This higher accuracy could be explained by the fact that an AT worn at the hip is closer to the body's center of mass, which would facilitate the detection of the whole body's acceleration. Figure 4 highlights significant correlations between the SC at the hip and the AER of SC in all samples of the population. This leads us to consider the Actigraph's accuracy with caution depending on its position. Figure 2 shows a good correlation between the SC of both Actigraph GT3X, but with poor ICC. In addition, the wide range in the limits of agreement ([−1,314; 9,987]) emphasizes the idea that the two device locations should not be used interchangeably without accounting for the existing bias when measuring the SC. In other words, an Actigraph GT3X worn at the wrist will tend to be less accurate than the same AT worn at the hip. The percentage of error can induce a significant difference in the SC according to the position of the AT.

Other factors may be involved in the significant SC differences observed between each position during activities in real-life conditions:

- The AT placed on the wrist records all accelerations of the forearm. During the day, an individual comes across a large number of activities which require the use of the upper limbs without the use of the lower limbs. As an example, while seated, one may actively move one's hands when eating, conversing, or when interacting with a screen, among other activities (29).
- According to Thielemans et al. (31), the better accuracy of wrist-worn devices at higher gait speed could be explained by differences in peak angular momentum. The peak angular momentum of arm swinging movements increases as walking speed increases, which causes greater changes in wrist velocity and facilitate its detection by a worn device.
- O'Connell et al. (32) showed that depending on the daily activities and the positioning of the AT, a certain number of positive false steps will be recorded. For example, the Jawbone™ AT positioned on the right wrist recorded steps while the subject was driving. This study also pointed out that positive false steps are observed in our study regardless of the Actigraph position.

### Practical Application

Our study shows that two identical Actigraph GT3X placed at the hip or at the wrist will generate a consequential AER in real-life situations. We were able to affirm that this error was multifactorial. In a study comparing the accuracy of the Actigraph GT3X and ActivPal, Steeves et al. (8), showed that a 4% difference in SC may amount to an error of 37 extra minutes of walking per day. Out of a 7-days observation period, the use of an AT with underestimation errors higher than 14% may translate into errors corresponding to more than one entire day of walking activity (30). As a reminder, the World Health Organization recommends 30 min of walking per day, 5 days a week. In our study, the average of the differences between the SC of the wrist vs. the hip is 30% higher in older adults compared to younger subjects (Table 2). These 30% represent a large part of their daily activity. In the light of our work, we argue that the accuracy of the sensors, which directly depends on the technology and processing algorithm, will have to be considered differently between young and old subjects or patients with motor disabilities. Indeed, elderly subjects tend to take fewer and slower steps, which strongly influences the variation between the estimated and real SC. Therefore, it seems essential to accurately identify the target population and the intended type of activity. Besides, the scientific literature clearly shows that a consistent use of the same AT is essential. A wide variety of models are indeed available on the market, and the SC obtained for a given activity is often different from one AT to another (33). Moreover, the position of the AT on the body seems to be an even more discriminating criterion of SC. Our work demonstrated that the difference between the SC measured at the wrist and hip can range from 30% in young subjects to nearly 50% in elderly subjects. The lack of accuracy in the measurement of the SC from an AT placed at the wrist may represent an issue for scientific uses, as it would reflect the number of movements performed by the upper limbs and not a real SC. It may however be a good indicator of a more global amount of PA. To obtain an optimal evaluation of the SC as close as possible to reality, placing the AT at the hip appears to be the most favorable position, with the exception of the ankle. Furthermore, a wide majority of activity tracking devices tend to underestimate the SC at slow speeds (<1 m.s^−1^) (2). The few AT models which are able to correctly identify slow steps are generally expensive (>$400) and unaffordable for the general public.

### Limitations of the Study

Our study may have been limited by its small population. However, our results are supported by the many results within the literature and provide a major complement to the use of Actigraph GT3X at the wrist or hip. Besides, as there is no gold-standard solution to evaluate the SC in free-living situations, our work does not allow us to assert which Actigraph GT3X provides the most precise values, i.e., the closest to reality. Further studies are therefore required to identify optimal AT placements at least for low-to-moderate activity, and in which position these monitors are mostly used by consumers during free-living conditions. In light of our study, it seems necessary to carefully consider the position of the AT, the age of the users and their lifestyle habits to achieve this objective.

## Conclusion

Our study showed that wearing the AT at the wrist may provide overestimated SC compared to the same AT model placed at the hip in young and elderly people in free-living conditions. On the one hand, this difference appeared to be accentuated according to the age of the subjects. On the other hand, it seems that the difference between the two positions tended to decrease for higher SC. These results suggest the hypothesis that the gait speed is an essential criterion when estimating the SC using an accelerometer. The assessment of the amount of PA in free-living conditions based on the SC remains uncertain and imprecise. The literature on the subject is extremely abundant and rather difficult to synthesize. Further work will be needed to improve the quality of SC measurement in free-living conditions for all populations (young, old, healthy or patient).

## Ethics Statement

All subjects gave written informed consent in accordance with the Declaration of Helsinki. The protocol was approved by the CERNI of the Univ. Grenoble Alpes, France.

## Author Contributions

SM: conceptualization, project administration, methodology, formal analysis, and writing original draft preparation. JL and AP: conceptualization, investigation, and methodology. ZS: validation. TA: formal analysis. NV: conceptualization, methodology, and formal analysis. All authors read and approved the final manuscript.

### Conflict of Interest

TA was employed by the company Orange Labs. The remaining authors declare that the research was conducted in the absence of any commercial or financial relationships that could be construed as a potential conflict of interest.

## References

[1] EUR/RC65/9 Physical Activity Strategy for the WHO European Region 2016–2025 - 65wd09e_PhysicalActivityStrategy_150474.pdf (2015). Available online at: http://www.euro.who.int/__data/assets/pdf_file/0010/282961/65wd09e_PhysicalActivityStrategy_150474.pdf (accessed January 17, 2019).

[2] Bassett DR Toth LP LaMunion SR Crouter SE. Step counting: a review of measurement considerations and health-related applications. Sports Med Auckl NZ. (2017) 47:1303–15. 10.1007/s40279-016-0663-1 28005190 PMC5488109

[3] Füzéki E Banzer W. Physical activity recommendations for health and beyond in currently inactive populations. Int J Environ Res Public Health. (2018) 15:E1042. 10.3390/ijerph15051042 29789470 PMC5982081

[4] Chowdhury EA Western MJ Nightingale TE Peacock OJ Thompson D. Assessment of laboratory and daily energy expenditure estimates from consumer multi-sensor physical activity monitors. PLoS ONE. (2017) 12:e0171720. 10.1371/journal.pone.0171720 28234979 PMC5325221

[5] Karabulut M Crouter SE Bassett DR. Comparison of two waist-mounted and two ankle-mounted electronic pedometers. Eur J Appl Physiol. (2005) 95:335–43. 10.1007/s00421-005-0018-3 16132120

[6] Storm FA Buckley CJ Mazzà C. Gait event detection in laboratory and real life settings: accuracy of ankle and waist sensor based methods. Gait Posture. (2016) 50:42–6. 10.1016/j.gaitpost.2016.08.012 27567451

[7] Magistro D Brustio PR Ivaldi M Esliger DW Zecca M Rainoldi A. Validation of the ADAMO Care Watch for step counting in older adults. PLoS ONE. (2018) 13:e0190753. 10.1371/journal.pone.0190753 29425196 PMC5806873

[8] Steeves JA Bowles HR McClain JJ Dodd KW Brychta RJ Wang J. Ability of thigh-worn ActiGraph and activPAL monitors to classify posture and motion. Med Sci Sports Exerc. (2015) 47:952–9. 10.1249/MSS.0000000000000497 25202847 PMC6330899

[9] Chow JJ Thom JM Wewege MA Ward RE Parmenter BJ. Accuracy of step count measured by physical activity monitors: the effect of gait speed and anatomical placement site. Gait Posture. (2017) 57:199–203. 10.1016/j.gaitpost.2017.06.012 28666177

[10] Feng Y Wong CK Janeja V Kuber R Mentis HM. Comparison of tri-axial accelerometers step-count accuracy in slow walking conditions. Gait Posture. (2016) 53:11–6. 10.1016/j.gaitpost.2016.12.014 28064084

[11] Grant PM Dall PM Mitchell SL Granat MH. Activity-monitor accuracy in measuring step number and cadence in community-dwelling older adults. J Aging Phys Act. (2008) 16:201–14. 10.1123/japa.16.2.201 18483442

[12] Simpson LA Eng JJ Klassen TD Lim SB Louie DR Parappilly B. Capturing step counts at slow walking speeds in older adults: comparison of ankle and waist placement of measuring device. J Rehabil Med. (2015) 47:830–5. 10.2340/16501977-1993 26181670

[13] Tudor-Locke C Barreira TV Schuna JM. Comparison of step outputs for waist and wrist accelerometer attachment sites. Med Sci Sports Exerc. (2015) 47:839–42. 10.1249/MSS.0000000000000476 25121517

[14] Crowley P Skotte J Stamatakis E Hamer M Aadahl M Stevens ML. Comparison of physical behavior estimates from three different thigh-worn accelerometers brands: a proof-of-concept for the Prospective Physical Activity, Sitting, and Sleep consortium (ProPASS). Int J Behav Nutr Phys Act. (2019) 16:65. 10.1186/s12966-019-0835-0 31419998 PMC6697962

[15] Kamada M Shiroma EJ Harris TB Lee I-M. Comparison of physical activity assessed using hip- and wrist-worn accelerometers. Gait Posture. (2016) 44:23–8. 10.1016/j.gaitpost.2015.11.005 27004628 PMC4806562

[16] Motl RW Sosnoff JJ Dlugonski D Suh Y Goldman M. Does a waist-worn accelerometer capture intra- and inter-person variation in walking behavior among persons with multiple sclerosis? Med Eng Phys. (2010) 32:1224–8. 10.1016/j.medengphy.2010.08.015 20875952 PMC3165016

[17] Webber SC St John PD. Comparison of ActiGraph GT3X+ and StepWatch step count accuracy in geriatric rehabilitation patients. J Aging Phys Act. (2016) 24:451–8. 10.1123/japa.2015-0234 26751505

[18] Chandrasekar A Hensor EMA Mackie SL Backhouse MR Harris E. Preliminary concurrent validity of the fitbit-zip and actigraph activity monitors for measuring steps in people with polymyalgia rheumatica. Gait Posture. (2018) 61:339–45. 10.1016/j.gaitpost.2018.01.035 29427859

[19] Moon Y McGinnis RS Seagers K Motl RW Sheth N Wright JA. Monitoring gait in multiple sclerosis with novel wearable motion sensors. PLoS ONE. (2017) 12:e0171346. 10.1371/journal.pone.0171346 28178288 PMC5298289

[20] Migueles JH Cadenas-Sanchez C Ekelund U Delisle Nyström C Mora-Gonzalez J Löf M. Accelerometer data collection and processing criteria to assess physical activity and other outcomes: a systematic review and practical considerations. Sports Med Auckl NZ. (2017) 47:1821–45. 10.1007/s40279-017-0716-0 28303543 PMC6231536

[21] Horvath S Taylor DG Marsh JP Kriellaars DJ. The effect of pedometer position and normal gait asymmetry on step count accuracy. Appl Physiol Nutr Metab. (2007) 32:409–15. 10.1139/H07-001 17510675

[22] Floegel TA Florez-Pregonero A Hekler EB Buman MP. Validation of consumer-based hip and wrist activity monitors in older adults with varied ambulatory abilities. J Gerontol A Biol Sci Med Sci. (2016) 72:229–36. 10.1093/gerona/glw098 27257217 PMC6082588

[23] Korpan SM Schafer JL Wilson KC Webber SC. Effect of ActiGraph GT3X+ position and algorithm choice on step count accuracy in older adults. J Aging Phys Act. (2015) 23:377–82. 10.1123/japa.2014-0033 25102469

[24] Cohen J Statistical Power Analysis for the Social Sciences. Hillsdale, NJ: Lawrence Earlbaum Associates (1988).

[25] Shrout PE Fleiss JL. Intraclass correlations: uses in assessing rater reliability. Psychol Bull. (1979) 86:420–8. 10.1037/0033-2909.86.2.420 18839484

[26] Bergman RJ Bassett DR Muthukrishnan S Klein DA. Validity of 2 devices for measuring steps taken by older adults in assisted-living facilities. J Phys Act Health. (2008) 5(Suppl. 1):S166–75. 10.1123/jpah.5.s1.s166 18364521

[27] Auvinet B Berrut G Touzard C Moutel L Collet N Chaleil D. Reference data for normal subjects obtained with an accelerometric device. Gait Posture. (2002) 16:124–34. 10.1016/S0966-6362(01)00203-X 12297254

[28] Bohannon RW Andrews AW Thomas MW. Walking speed: reference values and correlates for older adults. J Orthop Sports Phys Ther. (1996) 24:86–90. 10.2519/jospt.1996.24.2.86 8832471

[29] Aziz O Robinovitch SN Park EJ Identifying the number and location of body worn sensors to accurately classify walking, transferring and sedentary activities, in 2016 38th Annual International Conference of the IEEE Engineering in Medicine and Biology Society (EMBC) (Orlando, FL), 5003–6. 10.1109/EMBC.2016.7591851 28269392

[30] Storm FA Heller BW Mazzà C. Step detection and activity recognition accuracy of seven physical activity monitors. PLoS ONE. (2015) 10:e0118723. 10.1371/journal.pone.0118723 25789630 PMC4366111

[31] Thielemans V Meyns P Bruijn SM. Is angular momentum in the horizontal plane during gait a controlled variable? Hum Mov Sci. (2014) 34:205–16. 10.1016/j.humov.2014.03.003 24703335

[32] O'Connell S ÓLaighin G Quinlan LR When a step is not a step! Specificity analysis of five physical activity monitors. PLoS ONE. (2017) 12:e0169616 10.1371/journal.pone.0169616 28085918 PMC5234787

[33] An H-S Jones GC Kang S-K Welk GJ Lee J-M. How valid are wearable physical activity trackers for measuring steps? Eur J Sport Sci. (2016) 17:360–8. 10.1080/17461391.2016.1255261 27912681

